# Ecological divergence in the silver moss *Bryum argenteum*: developmental, ontogenetic and life‐history trait variations across contrasting tropical ecosystems

**DOI:** 10.1111/plb.70200

**Published:** 2026-03-11

**Authors:** W. L. dos Santos, A. Medina‐Ramos, J. Greenwood, K. C. Pôrto, F. Pinheiro, L. R. Stark

**Affiliations:** ^1^ Graduate Program in Ecology, Department of Plant Biology, Biology Institute Universidade Estadual de Campinas Campinas São Paulo Brazil; ^2^ School of Life Sciences University of Nevada Las Vegas Nevada USA; ^3^ Department of Botany, Biosciences Center Universidade Federal de Pernambuco Recife Pernambuco Brazil

**Keywords:** Adaptation, asexual reproduction, gametophyte development, geographical distance effect, plant reproduction, sex‐specific fitness, trade‐off

## Abstract

- This study investigates the reproductive and vegetative development of *Bryum argenteum* in two contrasting tropical environments: the Seasonal Tropical Dry Forest (SDTF) and the Brazilian Atlantic Forest (BAF). By comparing ecotypes from these regions, we aimed to understand how environmental variation influences sex‐specific traits, reproductive strategies and potential trade‐offs between sexual and asexual reproduction.
- We conducted temporal analyses of protonema growth, shoot production, sex expression, bulbil formation and gametangia development in laboratory‐grown samples representing male and female ecotypes from both environments.
- Significant differences were found between ecotypes and sexes. Male BAF ecotypes showed lower protonema growth and delayed shoot production compared to other groups. SDTF males exhibited the highest sex expression at Week 8. A negative relationship between sex expression and bulbil production was detected only in SDTF males. Gametangia development also varied, with male BAF ecotypes requiring more time to initiate sexual expression and mature phases revealing further distinctions.
- The observed differences across ecotypes and sexes reflect adaptive strategies shaped by the ecological conditions of each habitat. In particular, the delayed or reduced reproductive investment in male BAF ecotypes and the trade‐offs in females suggest context‐dependent allocation of resources. These patterns highlight the complex interplay between environment, sex and reproductive timing in tropical mosses.

## Graphical abstract

Ecotypes of the cosmopolitan moss *Bryum argenteum* differ markedly in growth, reproductive timing, and allocation across contrasting tropical ecosystems. These patterns reveal strong environment‐ and sex‐dependent life‐history strategies, highlighting the species as a model for studying ecological divergence in bryophytes.

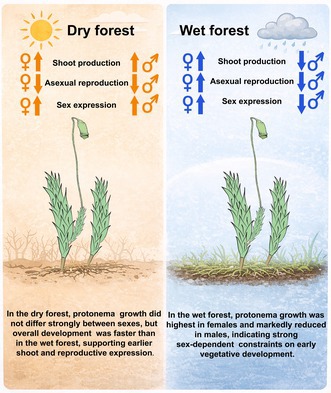

## INTRODUCTION

Reproduction stands as one of the most crucial features in the life cycle of plants, ensuring the maintenance of populations in ecosystems (Oli & Coulson 2016). Typically, plants reproduce sexually when the male gamete fertilizes the female gamete, yet asexual reproduction can also occur when clones are formed by vegetative propagules produced by the plant (Glime & Bisang 2017). These two reproductive modes play fundamental roles in the maintenance and structure of populations (Frey & Kürschner 2011).

The production of vegetative propagules can differ between sexes in dioecious plant species (dos Santos *et al*. 2024). This disparity stems from the reproductive cost associated with each sex (Obeso 2002), a consequence of substantial resource allocation in sexual reproduction (dos Santos *et al*. 2024). As sexes display different patterns of reproductive allocation, this leads to varying effects on other reproductive characteristics of plants (Ashman 1994; Bazzaz *et al*. 2005; Bisang *et al*. 2006). Concerning the reproductive mode of plants, some studies have shown resource competition between sexual and asexual reproduction, and this competition varies according to sex (Kimmerer 1991; Laaka‐Lindberg 2001; Fuselier & Mcletchie 2002; Van Drunen & Dorken 2012).

Differences between sexes in reproduction rate, vegetative propagule production and phenological development can significantly impact population parameters such as sex expression, sex ratio and population dynamics (Horsley *et al*. 2011; Eppley *et al*. 2018). For instance, species expressing only one sex might allocate more resources towards vegetative propagule production, increasing the likelihood of biasing the sex ratio towards that specific sex (McLetchie 1992; McLetchie & Puterbaugh 2000). Several studies have demonstrated that variations in development and reproduction can lead to changes in population structure, from sex distribution to genetic makeup (Wang *et al*. 2012; Bona *et al*. 2019; Yin *et al*. 2019). Moreover, these sex differences can vary on a geographical scale, characterizing populations according to the environment they inhabit (Bowker *et al*. 2000; Cascante *et al*. 2002; Castetter *et al*. 2019; Yin *et al*. 2019; Boquete *et al*. 2023).

Geographical distance influences microhabitat divergence due to varying environmental conditions, directly affecting the phenology and reproductive biology of species (Bisang *et al*. 2020; Boquete *et al*. 2023). When comparing two geographically distinct areas, factors such as latitude, altitude, climate and environmental conditions can vary considerably (Maciel‐Silva *et al*. 2012; Boquete *et al*. 2023). These environmental variations can result in diverse phenological schedules, including stages of flowering, fruiting and leaf fall (Satake *et al*. 2022). Additionally, these environmental variations can impact population demographics, including sex ratios, unveiling notable phenological and biological diversity among distinct regions (Boquete *et al*. 2023). Therefore, geographical distance can influence plant phenology, causing temporal variations in growth and reproductive phases across different areas. For example, in species where sex differences exist, such as in the case of *Syntrichia caninervis* Mitt., male and female plants express their sexes under different environmental conditions (Bowker *et al*. 2000). Thus, understanding the relationship between the environment and an organism's biology is crucial for comprehending how these organisms adapt to diverse conditions (Leimu & Fischer 2008).

Plants are suitable organisms for studying the impact of contrasting environmental conditions on the biology of organisms. Due to their sessile nature, plants offer greater ease of handling during experiments (Szczepaniak & Biziuk 2003). Among terrestrial plants, bryophytes, a monophyletic group encompassing mosses, liverworts and hornworts (Harris *et al*. 2020), have been considered more advantageous as ecological models compared to vascular plants. Primarily, this preference stems from their reproductive cycle, characterized by an alternation between haploid generations (gametophyte, where male – antheridia – and female – archegonia – gametes form) and diploid generations (sporophyte), where the gametophyte dominates and the sporophyte relies on the gametophyte for development. Secondly, by being morphologically simple and smaller compared to angiosperms, bryophytes small, exhibit high regenerative capacity and possess broad geographical distribution (McLetchie 1992; Bisang & Ehrlén 2002; Moore *et al*. 2016).

Species with broad geographical distributions provide valuable opportunities to explore the life‐history traits in response to environmental differences (Pannell *et al*. 2014; Boquete *et al*. 2023). The silver moss, *B. argenteum* Hedw., serves as a typical example within the moss family Bryaceae (Shaw & Albright 1990). This species is cosmopolitan, found across all continents and within diverse ecosystems (Longton 1981; Pôrto *et al*. 2017; Gabriel *et al*. 2019; Yuqing *et al*. 2021). Furthermore, *B. argenteum* possesses a dioicous sexual system, rendering it particularly suitable for comparative analyses between sexes. In this study, we employed *B. argenteum* to investigate differences in development, ontogeny and life‐history traits associated with sexes (male and female) and two different ecotypes present in two contrasting environments. The first forest is the Brazilian Atlantic Forest (BAF), renowned for its low seasonality climate and high precipitation levels. On the other hand, the second forest is the Seasonal Dry Tropical Forest (SDTF), characterized by highly seasonal conditions and low rainfall (Alvares *et al*. 2013). We investigate the following questions:

1. Are there variations in protonema growth rate and shoot production between sexes and distinct ecotypes?
2. Does the timing of protonema development, shoot emergence and sexual expression differ between sexes and ecotypes of *B. argenteum*?
3. Are there variations in sex expression among the analysed sexes and distinct ecotypes?
4. Does a trade‐off exist between sexual reproduction (gametangia production) and asexual reproduction (bulbil production)?
5. Are there disparities in gametangia development between sexes and studied ecotypes?

## MATERIAL AND METHODS

### Studied species

*Bryum argenteum* is characterized by the silver colour when dry, caused by the absence of chloroplasts in the leaves' upper region, making them hyaline (Beever 1992). Being a dioicous species, the production of male and female gametangia occurs in separate plants (Liang *et al*. 2010). Antheridia (male gametangia) are found at the tip of the male shoot, and archegonia (female gametangia) at the base of the female shoot, mostly (Liang *et al*. 2010). Bulbil (asexual reproduction) is also commonly found in shoots. In Brazil, *B. argenteum* is found in all ecosystems, from the driest and hottest to the wettest and coldest (Costa & Peralta 2015). *B. argenteum* has a long history in terms of biology, related to the reproductive biology (Chopra & Rawat 1977; Horsley *et al*. 2011; Pôrto *et al*. 2017), tolerance to desiccation (Proctor *et al*. 2007; Stark *et al*. 2016; Yuqing *et al*. 2021) and high‐temperature tolerance (Zhuo *et al*. 2020). This is because the species is favourable to the stabilization and growth of colonies in the laboratory, favouring the performance of experiments.

### Study site, sample and growth

**Fig. 1:**
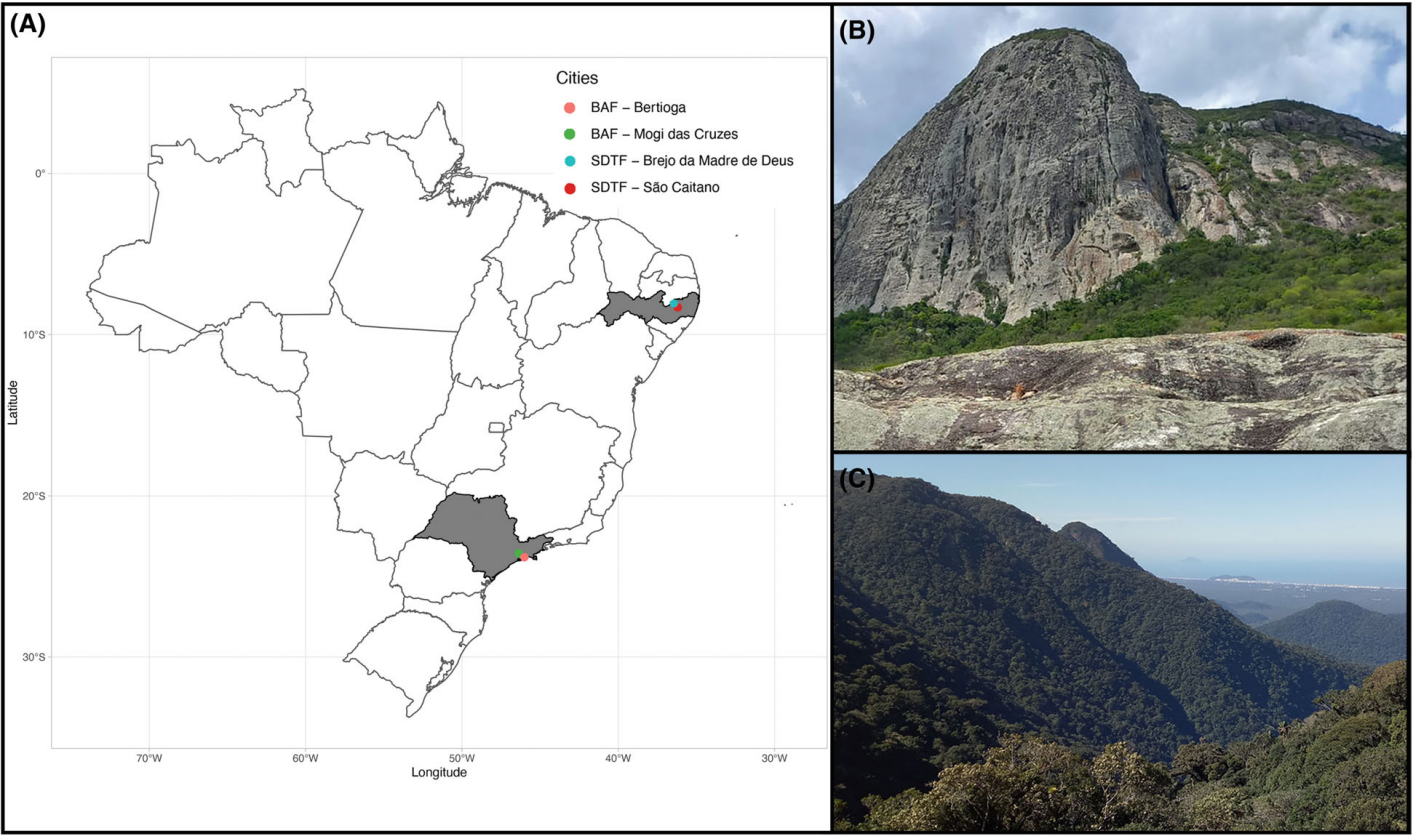
(A) Brazilian map with points collected in SDTF and BAF. (B) View of Private Natural Heritage Reserve Pedra do Cachorro (SDTF) and (C) view of Parque das Neblinas (BAF).

We sampled *B. argenteum* in two contrasting Neotropical biomes. The first was the Seasonal Dry Tropical Forest (SDTF or Caatinga; Fig. 1), an exclusively Brazilian biome with vegetation adapted to xeric conditions (de Souza *et al*. 2015). SDTF climates show 300–1000 mm of annual rainfall concentrated in 3–5 months and temperatures of 25–30 °C (de Queiroz *et al*. 2018). Samples were collected in two Pernambuco reserves: RPPN Pedra do Cachorro (São Caitano; 8°14′12.8″ S, 36°11′33.2″ W) and RPPN Brejo da Madre de Deus (8°08′40.2″ S, 36°21′42.9″ W). The second biome was the Brazilian Atlantic Forest (BAF), one of the most species‐rich biomes in the country (Costa & Peralta 2015). We sampled at Parque das Neblinas (Mogi das Cruzes and Bertioga; 23°44′13.0″ S, 46°10′37.7″ W), where rainfall averages 2400 mm year‐round and temperatures range from 16 to 18 °C (Onofre *et al*. 2010). In total, 10 populations were sampled, five per biome. From each population, one male and one female shoot expressing sexual organs were selected, clonally propagated once to standardize environmental history and then used to generate five clonal replicates per sex per ecotype (100 Petri dishes).

Colonies of approximately 100 cm^2^ were sampled from each population. The populations were collected with a minimum distance of 100 m separation. This distance was established based on the observation that several sporophytic colonies containing male shoots did not influence the fertilization of female shoots in colonies expressing sexual characteristics when they were located at distances <10 m. Therefore, we assumed that this distance would ensure the presence of shoots with distinct ecotypes. The samples were preserved in paper bags and kept at room temperature (approx. 20 °C). Species identity was confirmed following Canestraro & Peralta (2022). After specimens' confirmation, shoots that expressed sex (*i.e*., shoots that had perigonia and perichaetia) were identified and separated. Thus, one male and female were selected for each population and accuminated separately to be clonally propagated. Then, all the material used for the cultivation was prepared. The sand was collected in Las Vegas, NV, USA, near Red Rock Canyon National Conservation Area, sieved through a 350 μm mesh and autoclaved for 60 min at 121 °C. The use of sterilized sand as a growth substrate follows established protocols successfully applied in previous studies with *B. argenteum* and other moss species (Horsley *et al*. 2011; Stark & Brinda 2013). This inert and homogeneous substrate allows for standardized growing conditions across replicates, eliminating the variability in physicochemical properties often found in native soils. Consequently, this approach facilitates the isolation of biological responses from environmental influences, ensuring reproducibility and comparability across experiments. As well as the sand, all materials used for the cultivation were autoclaved. After autoclaving, approximately three scoops of sand were placed in 20 Petri dishes of 35 mm diameter. Then they were hydrated with about 12–15 drops of water.

After the Petri dish preparation, the separated shoots were prepared for cultivation. First, with a stereomicroscope and fine‐tipped tweezers, the shoot was analysed and cleaned in water on a slide, removing all dirt and soil residue. In each new washing of the shoot, the tweezers were cleaned with 70% alcohol. Once cleaned, the tip was separated from the shoot and cleaned again in water. Subsequently, the tip was washed for 5 s in a 2% sodium hypochlorite solution (conventional bleach). After 5 s, the tip was placed in the centre of the Petri dish previously prepared with sand and water. Each Petri dish was identified with adhesive tape at the bottom and a permanent marker at the top. Once the Petri dishes were all composed of the tips, they were placed in a germination chamber at a temperature of 20 °C and constant light with an intensity of 100 to 410 μmol m^−2^ s^−1^ photosynthetically active radiation. The Petri dishes were hydrated with water twice a week, and samples were changed in position in the growth chamber.

After 2 months of culture, when all dishes had developed shoots, each ecotype was clonally propagated five times (in other words, each ecotype was replicated five times). Thus, totalling 100 Petri dishes, 25 for each sex (male and female) of each ecotype (SDTF and BAF). All tips placed to regenerate were washed for 5 s in a 2% sodium hypochlorite solution. All dishes were placed in a germination chamber at a temperature of 20 °C and constant light with an intensity of 100 to 410 μmol m^−2^ s^−1^ photosynthetic active radiation. Each Petri dish was hydrated twice a week, and once a week, each plate was photographed to follow the regeneration development. In the first month, the dishes were hydrated with distilled water. After the first month, hydration was alternated weekly between distilled water and 30% Hoagland solution.

### Phenological observations

#### Protonema growth and shoot production

With photos taken weekly, the area occupied by the protonema and the number of shoots produced was quantified for 8 weeks. After 8 weeks, quantifying all shoots became problematic, as the larger shoots covered the smaller ones and thus made exact quantification impossible. Therefore, we quantified shoot production only until Week 8. After Week 8, only the shoots that expressed sex were quantified. For the growth of the protonema, in the first 2 weeks, pictures were taken under a stereomicroscope at 40× magnification. From the third week onwards, the photos were taken at 6.3× magnification, as the area produced by the protonema exceeded the visual limit of 40× magnification. The photos were captured with a Nikon DSLR camera, model D780, attached to a Leica trinocular stereomicroscope.

Furthermore, with the lowest available magnification of 6.3×, it was possible to take a picture of the entire area of the Petri dish. The ImageJ software (Rueden *et al*. 2017) was used to quantify the area occupied by the protonema and the production of shoots. Photos of scales in mm were taken at each magnification photographed, which were later used for software calibration to calculate the area of protonema produced. Using the ImageJ software's Freehand selection tool, after the scale calibration, the area occupied by the protonema in the Petri dish was delimited, followed by the quantification of the protonema area. Finally, the day the protonema began to grow for each Petri dish was noted.

We used the ImageJ software particle analyser tool to quantify the production of shoots. The photos were edited, and image parameters such as contrast, lighting and shading were adjusted to leave the shoots with a more distinct colour from the soil and protonema. These images were transformed into eight bits and adjusted to black and white so that the shoots were highlighted, forming black points. With the black points (apex of each shoot), they were analysed and quantified by the particle analysis tool. For each Petri dish, the day the shoots began to be produced was noted.

#### Growth rate of protonema and shoot production

The protonema growth rate and shoot production were calculated for each colony using the following formula; $\text{rate of growth}\;\left(\mathrm{RG}\right)=\frac{\sum \left(\text{weekly growth}\right)}{\text{number of weeks}}$, which represents the average weekly growth over the course of the weeks. For protonemal growth, the area grown by the protonema was quantified each week. For shoot production, the number of shoots produced per week was counted. The obtained values were then divided by 8.

#### Sex expression

Weekly, sex expression was quantified in absolute terms (number of shoots that expressed sex per dish) and relative terms (proportion of shoots that expressed sex per dish). Relative sex expression was quantified up to 8 weeks since, after that time, it was not possible to quantify all shoots without sex expression due to population density. At the end of the observation period, we quantified the time for the beginning of protonema development, shoot production and sex expression for each dish separately. Absolute sex expression was quantified until Week 10.

#### Sexual reproduction *versus* bulbil production

Since the number of bulbils varied between ecotypes, we investigated possible trade‐offs between sexual and asexual reproduction. For this, we quantified the bulbils produced for each dish and the number of shoots that expressed the sex. Only the bulbils that were detached from the shoots were quantified. To quantify the bulbils, we hydrated the plates and then quantified them. This was done because, when hydrated, the bulbils float, facilitating their quantification. The bulbils were quantified in Week 8, as this was the week in which it was possible to quantify all the shoots (both expressed and unexpressed), making it possible to investigate a potential trade‐off between sexual reproduction and bulbil production.

#### Gametangia development

**Fig. 2:**
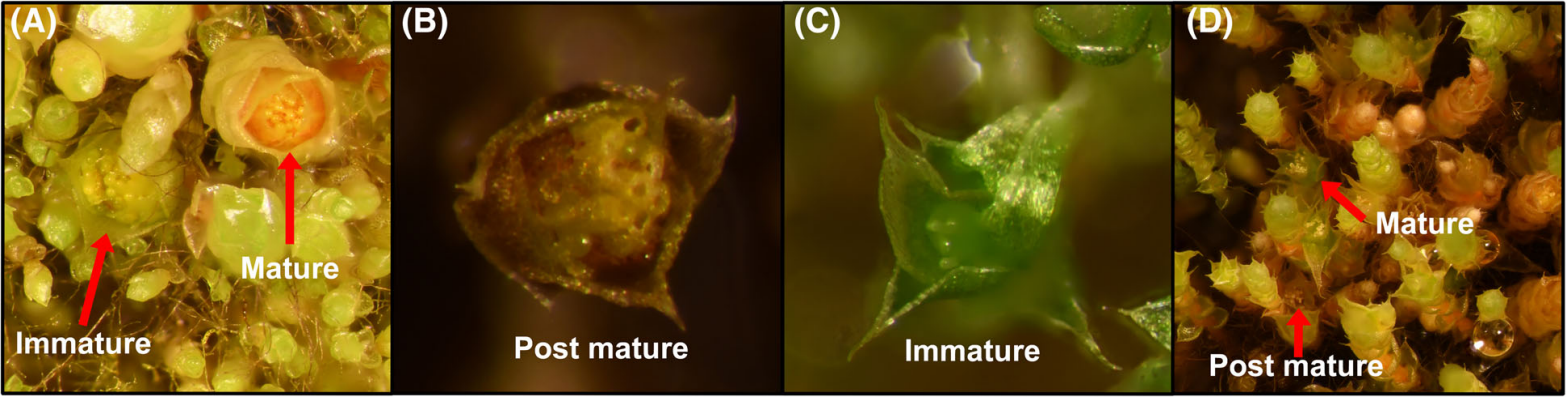
Male and female gametangia with their respective phenophases analysed in this study. (A) male perigonia mature (yellowish antheridia) and immature (green antheridia); (B) post‐mature perigonium (antheridia opened and brownish); (C) immature perichaetium (archegonia green and closed); (D) Perichaetia mature (open archegonia with colours yellowish and green) and post‐mature (open archegonia with brownish colour).

Regarding the development of gametangia, we classified and quantified the phenophases of gametoecia in three phases, namely: (i) immature – when the gametangia are intact and green, that is, closed apex; (ii) mature – when the male gametangia are yellowish and/or not releasing sperm and the female gametangia with one or more open and receptive apex; and (iii) post‐mature – when the gametangia are brownish and withered (Fig. 2). Weekly, for 10 weeks, the number of shoots that expressed sex and their respective phenophases were quantified, to monitor the development time of reproductive structures.

### Statistical analysis

#### Protonema growth and shoot production

To investigate our first question regarding potential differences in protonemal growth and shoot production, we employed Kruskal–Wallis analysis. For each analysis, protonemal growth and shoot production were used as dependent variables, while sex and ecosystem were included as explanatory variables. Initially, we assessed the normality and homoscedasticity of the data to determine the suitability of applying Analysis of Variance (ANOVA). However, as the data did not exhibit normal distributions even after transformations, we opted for Kruskal–Wallis to compare protonemal growth and shoot production across different sexes and ecosystems. For subsequent comparison, we applied Dunn's test with Bonferroni correction. The Dunn test was chosen because it is the appropriate *post hoc* procedure for non‐parametric analyses, allowing pairwise comparisons while controlling for Type I error when adjusted with Bonferroni (Dinno & Dinno 2017).

#### Beginning of protonema, shoot and sex expression

To investigate our second question, we employed the Generalized Linear Model (GLM) to compare the onset of protonema production, shoot production and sexual expression between the sexes and ecotypes under study. We created three models, utilizing ecotypes and sexes as predictor variables and the weeks of protonema initiation, shoot production and sex expression as response variables. The interactions of predictor variables were tested in all models. Each model was then compared using the chi‐square test against the equivalent null model (with the same response variable). Both the complete and null models were specified with the Poisson error family and the log‐link function. Subsequently, we checked the data dispersion and observed that all models showed a dispersion inconsistent with the model. Therefore, we adjusted the test family to quasipoisson. We performed the Sidak test for *post hoc* analysis, setting an alpha level of 0.05. We assessed model assumptions by examining diagnostic plots, including residuals *versus* fitted values, normal Q–Q plots, scale‐location plots and residuals *versus* leverage. These visual inspections did not reveal evidence of heteroscedasticity, deviations from residual normality or influential outliers. Thus, we considered the assumptions of the quasipoisson models to be reasonably met (see File S1).

#### Final absolute and relative sex expression rate

To address our third question, we compared Absolute sex expression at Week 8, Relative sex expression at Week 8 and Absolute sex expression at Week 10, each treated as a response variable. The predictor variable was the sex concatenated with ecotype (female BAF, male BAF, female SDTF, male SDTF). Because none of the response variables met the assumptions of normality, even after data transformations, we used the Kruskal–Wallis test to evaluate differences among the four groups. When the test was significant, we applied a Dunn test with Bonferroni correction.

#### Sex expression *versus* bulbil production

To address our fourth question, we analysed bulbil production (response variable) as a function of absolute and relative sex‐expression biomass (predictor variables), while also accounting for the four sex–ecotype groups: male BAF, female BAF, male SDTF and female SDTF. We fitted two negative binomial generalized linear models: (i) a model in which absolute sex expression at Week 8 and the four‐level sex variable were used as predictors, and (ii) a model in which relative sex expression at Week 8 and the four‐level sex variable served as predictors. For both analyses, we compared the full models against their respective null models using likelihood‐ratio tests. Model assumptions were evaluated through standard diagnostic plots. These checks indicated no problematic residual patterns, no signs of overdispersion and an appropriate mean–variance structure under the negative binomial distribution.

#### Gametangia development

To address the fifth question regarding possible differences in the development of gametangia, we chose to use the Kruskal–Wallis test due to the absence of a normal distribution in the data. In this analysis, the response variables were the week of first occurrence and the peak week of each phenophase (immature and mature), and the explanatory variable was the combined sex–ecotype group (male BAF, female BAF, male SDTF and female SDTF). In this context, we quantified the week of initial occurrence and the peak week (representing the moment when the highest quantity of the respective phenophase occurred) for both immature and mature phases within each group. To handle zero values, they were treated as missing data. This approach was adopted to avoid distortions in the temporal analysis since assigning a value of 0 would imply that the phenophase occurred instantly. By replacing missing values with the mean, we could more accurately represent the occurrence of the phenophase and its peak week without compromising the integrity of the data. After the Kruskal–Wallis analysis, the Dunn test was used for *post hoc* comparisons between groups, adjusted with Bonferroni correction to eliminate Type I error. The post‐maturation phase was excluded due to limited observations, with our analysis focused on the early stages of phenological development.

All statistical analyses were conducted using R version 4.2.1 (RStudio Team 2021). For data visualization and *post hoc* testing, we used the ggplot2 package version 3.5.1 (Wickham *et al*. 2016), the plotrix package version 3.8.2 (Lemon *et al*. 2009) and the dunn.test package version 1.3.5 (Dinno & Dinno 2017). Image analysis was performed using ImageJ2 software version 2.9.0/1.53t (Rueden *et al*. 2017), which was used for protonema area measurements and shoot quantification. These specific software versions are reported to ensure reproducibility and account for possible variation in functions across updates.

## RESULTS

### Protonema and shoot growth

**Fig. 3:**
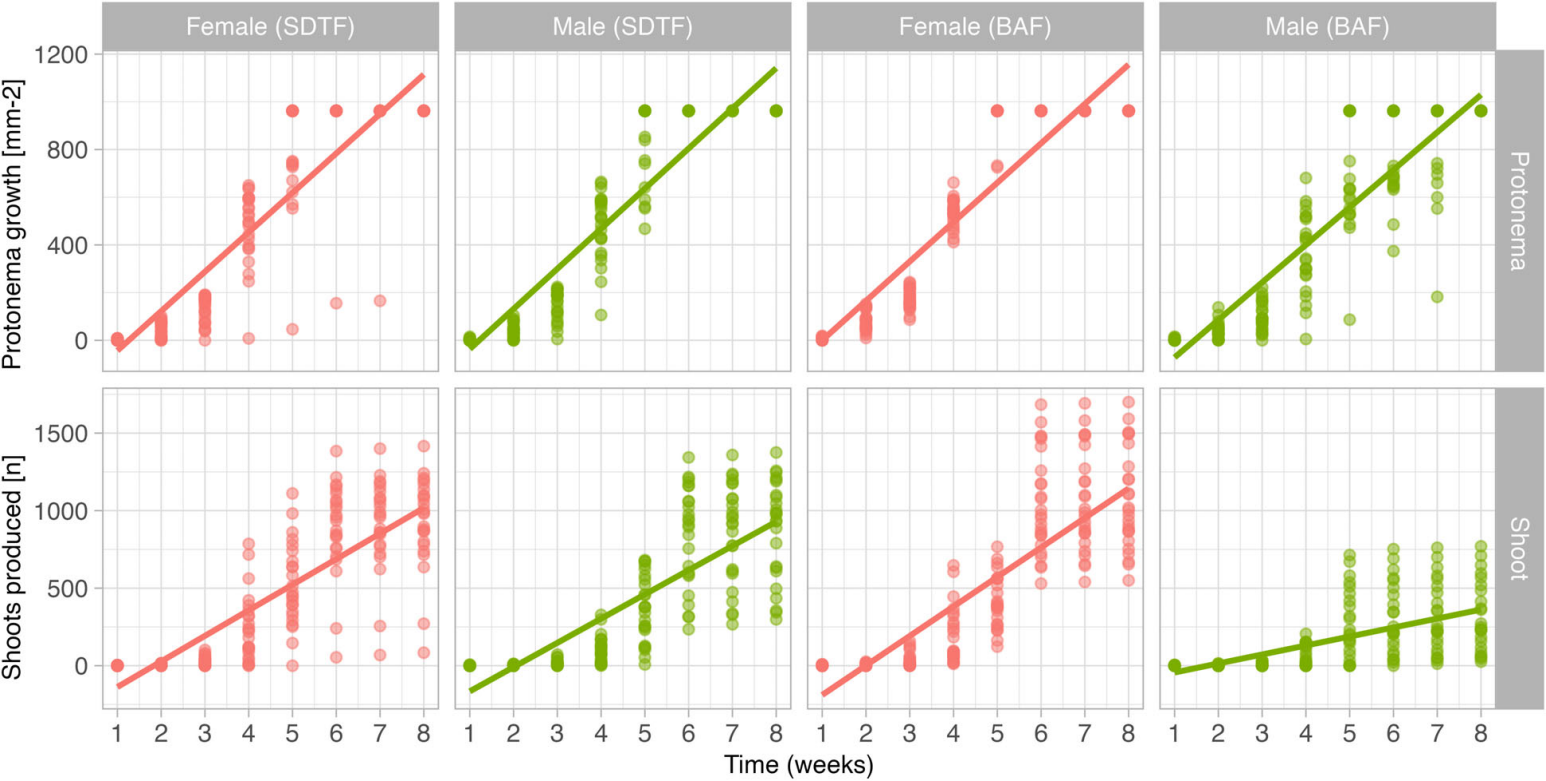
Scatterplots arranged in two rows: the first row displays the protonema area growth (mm‐^2^) over time (weeks), while the second row represents shoot production (n) over time (weeks). The columns show sexes per forest. Points on the plot represent colonies cultured in the laboratory, while the drawn line depicts the growth trend. Coral colour represents the female sex, and green colour represents the male sex.

The Kruskal–Wallis analysis regarding the protonemal growth rate revealed a highly significant result (Kruskal–Wallis test, χ^2^ = 18.87, df = 3, *P* < 0.001). In pairwise comparisons, female plants from BAF exhibited a significantly higher mean than female plants from SDTF. Additionally, the mean of female plants from BAF was greater compared to male plants from the same region. These higher means in the groups indicate that they grew more weekly (Fig. 3). The remaining groups did not show significant differences between their means. The Kruskal–Wallis analysis applied to compare shoot production rate also demonstrated a highly significant value (Kruskal–Wallis test, χ^2^ = 44.38, df = 3, *P* < 0.001). In pairwise comparisons, male plants from BAF displayed a significantly lower mean compared to the other groups: female plants from BAF and SDTF, and male plants from SDTF. These latter ones, in turn, did not show significant differences among themselves (Fig. 3).

### Beginning of protonema, shoot and sex expression

**Fig. 4:**
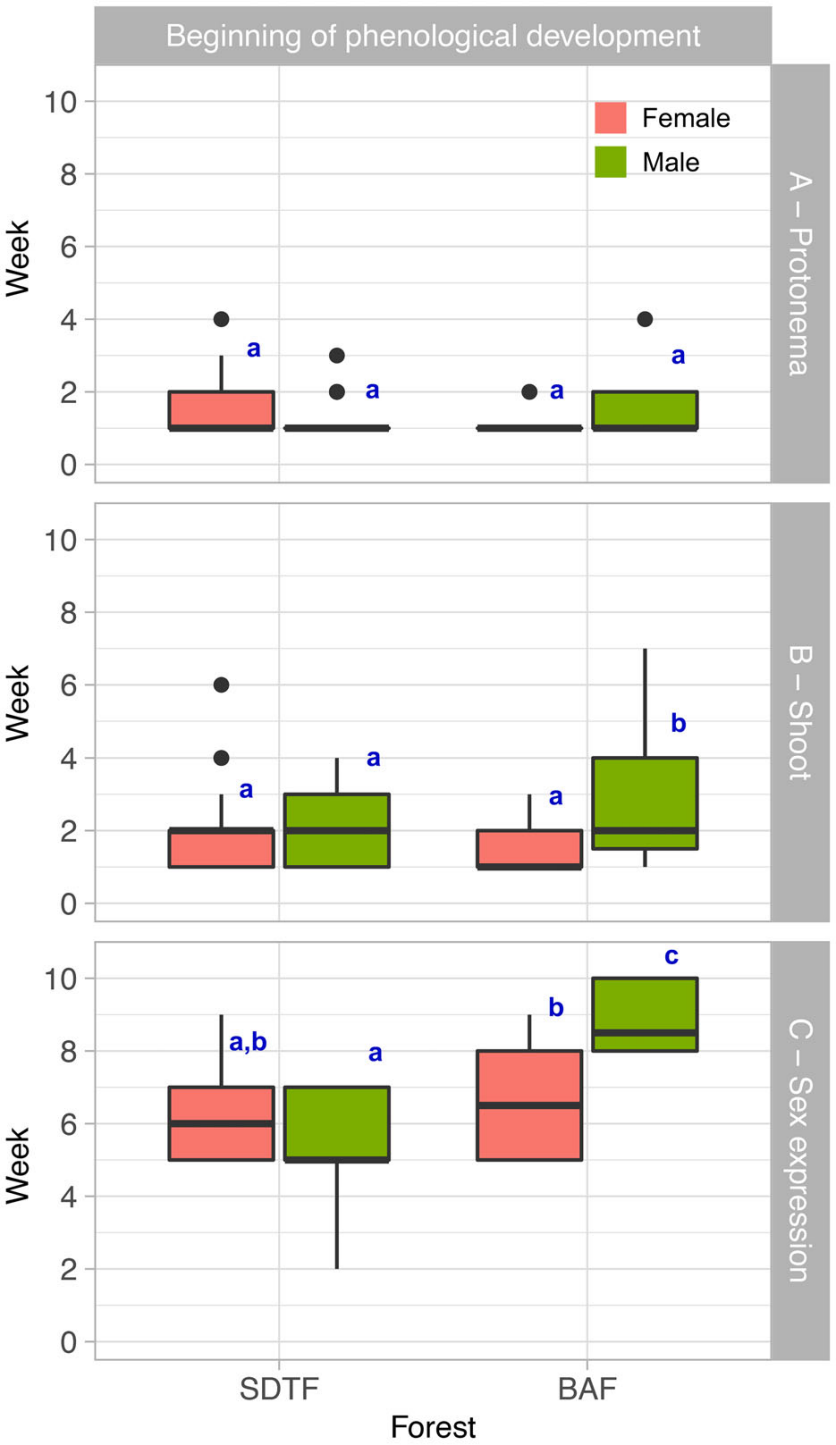
Boxplot displays the onset of development (time taken to initiate development of these traits) of protonema, shoots and sex expression for male and female colonies of BAF and SDTF varieties. The legend below the plot indicates the significance parameter between the groups, based on the *post hoc* test result of the generalized linear model (GLM). The coral colour represents female, and the green colour represents male.

The beginning of protonema production did not differ between the ecotypes and sexes studied (GLM, df = 3, Dev = 1.37, *P* = 0.71) (Fig. 4). However, shoot production exhibited a significant difference only in the interactions between predictor variables, not in the isolated predictor variables (GLM, df = 3, Dev = 14.80, *P* < 0.001). In this sense, the BAF male ecotype showed a significantly longer time for the start of shoot production when compared to the other sexes and ecotypes (Fig. 4). The beginning of sex expression differed between ecotypes (GLM, df = 3, Dev = 14.95, *P* < 0.01); male and female ecotypes from the SDTF had a shorter time for sex expression and did not differ from each other. The female BAF and SDTF ecotypes did not differ in time for the sex expression. However, the female BAF ecotypes had a significantly longer time to begin the sex expression when compared to the male SDTF ecotypes. The male ecotypes from the SDTF exhibited the longest duration of sex expression, distinguishing this ecotype from all the others under investigation (Fig. 4).

### Final absolute and relative sex expression rate

**Fig. 5:**
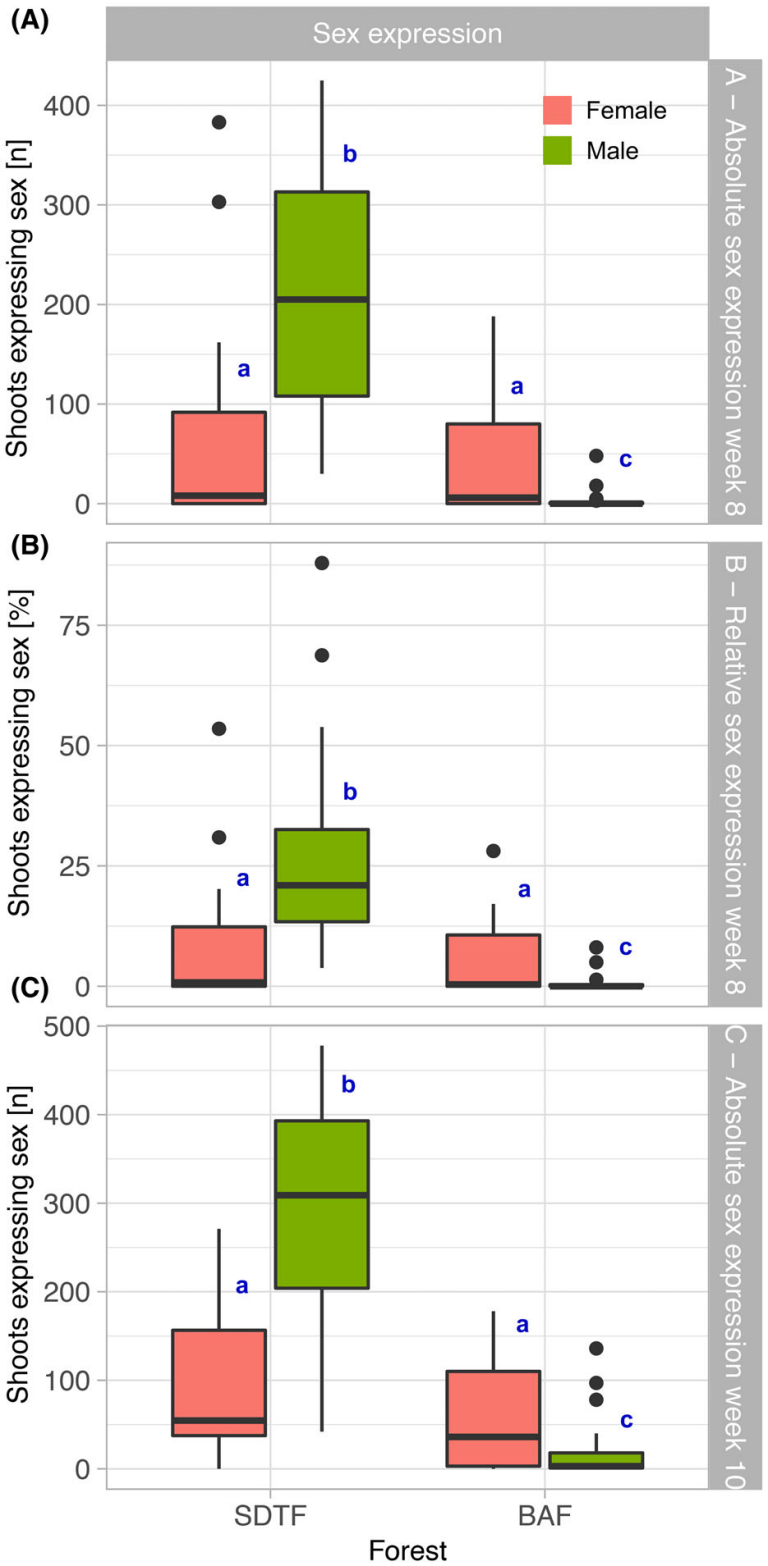
Boxplot displays: (A) absolute sex expression at Week 8; (B) relative sex expression at Week 8; (C) absolute sex expression at Week 10, for male and female cultures of BAF and SDTF varieties. The legend below the plot represents the significance parameters generated by the *post hoc* test of the generalized linear model (GLM). The coral colour represents female, and the green colour represents male.

Regarding the absolute sex expression rate at Week 8, the analyses showed significant differences between the sexes (Kruskal–Wallis test, χ^2^ = 49.27, df = 3, *P* < 0.001). Absolute sex expression at Week 8 was higher in SDTF males, while female shoots (SDTF and BAF) did not differ (Fig. 5). The male shoots from the BAF had the lowest average, differing from all ecotypes of SDTF and BAF females (Fig. 5). The analysis of relative sex expression (Week 8) also showed significant differences (Kruskal–Wallis test, χ^2^ = 47.38, df = 3, *P* < 0.001), with male shoots from the SDTF having the highest average compared to the other categories. The female shoots (BAF and SDTF) did not differ. BAF male shoots differed from all sexes (Fig. 5). Finally, absolute sex expression at Week 10 showed a significant difference (Kruskal–Wallis test, χ^2^ = 52.61, df = 3, *P* < 0.001). The difference followed the same pattern observed in comparing the sexes about relative sex expression (Fig. 5).

### Sex expression *versus* bulbil production

The negative binomial model using absolute sex expression as the predictor showed that the full model provided a significantly better fit than the null model (χ^2^ = 18.15, *P* = 0.001). However, absolute sex‐expression biomass itself was only marginally non‐significant, indicating a weak negative trend between absolute sex expression and bulbil production (Estimate = −0.0026, *P* = 0.06). Within this model, most sex‐group contrasts were not significant. The only group showing a clear tendency was SDTF males, which produced fewer bulbils compared to the reference category (Estimate = −0.713, *P* = 0.05), although this effect was marginal. Female BAF and female SDTF shoots did not differ significantly from the reference group. Diagnostic checks showed no overdispersion or residual problems, confirming good model fit (see File S2). The negative binomial model using relative sex expression also outperformed the null model (χ^2^ = 15.67, *P* = 0.001), but relative sex expression did not predict bulbil production (Estimate = −0.0064, *P* = 0.48). In this model, the only significant predictor was the SDTF male group, which produced substantially fewer bulbils than the reference category (Estimate = −1.0227, *P* < 0.01). All other contrasts, including both female groups and BAF males, were non‐significant. As in the previous analysis, model diagnostics indicated no overdispersion and no problematic residual structure.

### Gametangia development

The initial stage of gametangia development (immature phenophase) exhibited noticeable variations among the examined sexes and ecotypes (Kruskal–Wallis test, χ^2^ = 37.27, df = 3, *P* < 0.001). Males from the BAF group had a significantly longer mean time for initial sex expression compared to males and females from the SDTF and females from the BAF. However, there was no significant difference between males and females from the SDTF, as well as females from the BAF. Regarding the peak of the immature phenophase, significant differences were found among the compared groups (Kruskal–Wallis test, χ^2^ = 27.50, df = 3, *P* < 0.001). Shoots from the male BAF group significantly differed with a longer mean time compared to shoots from the female BAF and male SDTF groups. Additionally, shoots from the male SDTF group differed significantly from shoots from the female SDTF group. No significant differences were observed among the remaining groups.

**Fig. 6:**
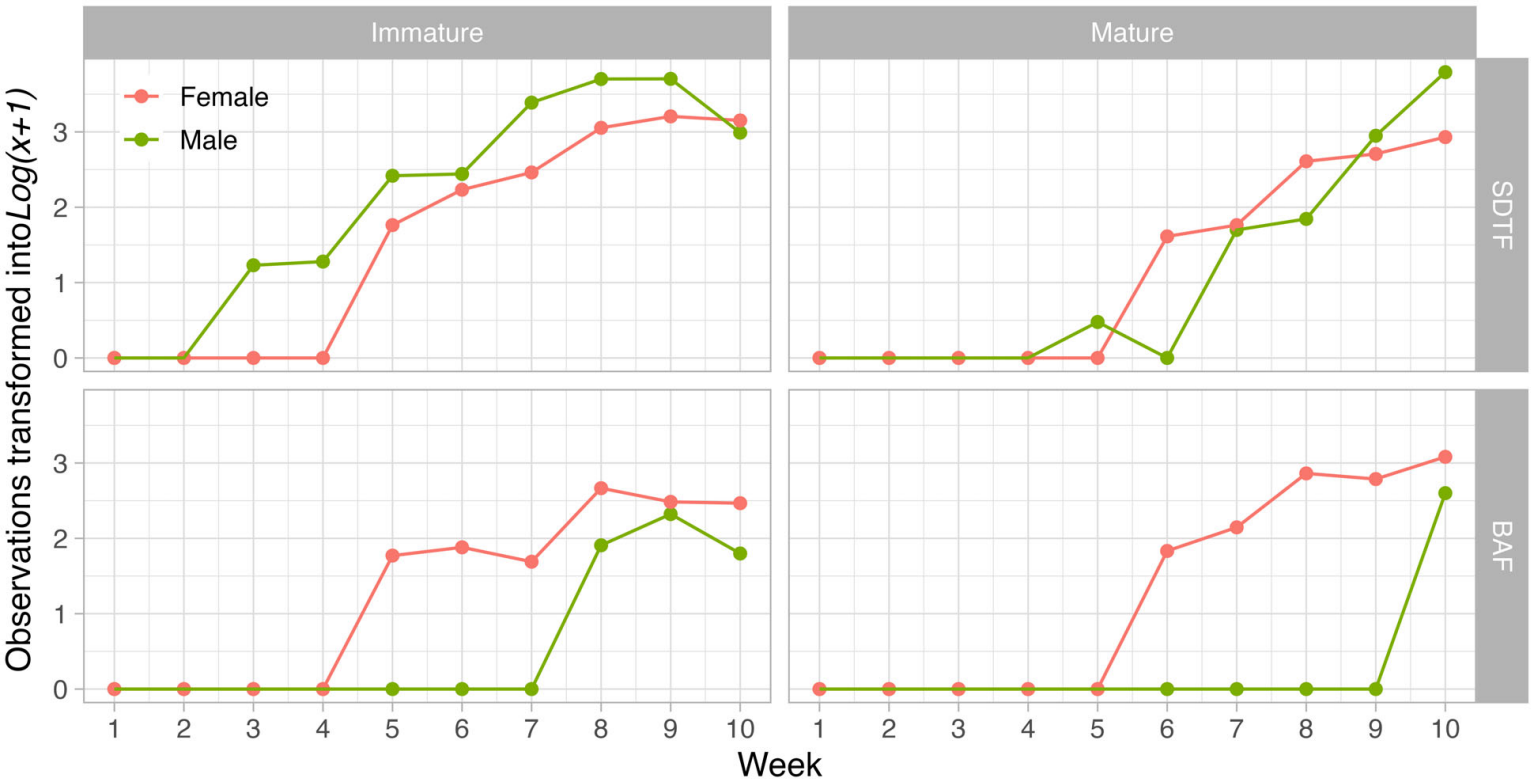
Point plot representing the immature and mature phenophases for male and female cultures of both studied ecotypes (SDTF and BAF). The y‐axis represents the total observations made each week, as depicted on the x‐axis. The data has been transformed using a log(x + 1) for better observation.

In relation to the mature phenophase, the test revealed a significant difference in the results (Kruskal–Wallis test, χ^2^ = 23.9, df = 3, *P* < 0.001). Male shoots from the BAF group had a longer mean time compared to female shoots from the BAF group, as well as both male and female shoots from the SDTF group. Additionally, there was a significant difference between male and female shoots from the SDTF group, with males from this group exhibiting a shorter mean time. No significant differences were found among the other groups. The test also indicated a significant difference in the results for the peak of the mature phenophase (Kruskal–Wallis test, χ^2^ = 13.58, df = 3, *P* < 0.001). Male shoots from the BAF group exhibited a shorter mean time compared to female shoots from the BAF group, as well as both male and female shoots from the SDTF group. There was no significant difference between female shoots from the BAF group and the other groups (Fig. 6).

## DISCUSSION

### The ecotypes show differences in growth parameters and suggest competitiveness between the sexes

Growth parameters of *B. argenteum* varied among the different sexes and ecotypes (Fig. 3). However, male shoots from the BAF tended to present low protonema growth and shoot production. These results show that shoot regeneration in *B. argenteum* is inconsistent and sex‐dependent, with distinct responses within the same species. For instance, Horsley *et al*. (2011) found differences in the rate of protonema growth and shoot production in some populations of *B. argenteum* collected in distinct environments, while other ecotypes did not differ in such traits. In the dioicous moss *Pleurozium schreberi* (Willd. ex Brid.) Mitt., Longton & Greene (1979) reported no sex differences in several reproductive traits, including vegetative growth rate, a pattern consistent with our SDTF results. Other species show clear sex‐specific contrasts: in *Polytrichum commune* Hedw., males produce more shoots than females (Wyatt & Darda 1997). In contrast, females have the advantage in *Pogonatum dentatum* (Brid.) Brid. (Hassel *et al*. 2005) and *S. caninervis*, where female shoots regenerate faster (Stark *et al*. 2005; Stark & McLetchie 2006). Likewise, female shoots of *Weissia jamaicensis* (Mitt.) Grout produce more axes (dos Santos *et al*. 2022b).

This faster development in female shoots is strongly related to the reproductive cost of the plants (Obeso 2002; Karlsson & Méndez 2005). Considering the pattern of reproductive allocation in bryophytes (greater allocation in male functions in rhizautoicous and dioicous systems at the prezygotic level), this generates a limitation of resources in male shoots to other vital functions of shoots (McLetchie & Puterbaugh 2000; Stark *et al*. 2000; Hedderson & Longton 2008; Horsley *et al*. 2011; Stark & Brinda 2013; dos Santos *et al*. 2018; dos Santos *et al*. 2022a; dos Santos *et al*. 2024). In *B. argenteum*, the prezygotic reproductive allocation is significantly higher in males compared to females reported by Horsley *et al*. (2011). This favourable reproductive allocation to male function entails the reproductive cost. Thus, a good development for female shoots is expected at the prezygotic level. Still, our findings indicate that populations of *B. argenteum* behave differently according to the environment where they are found. Indeed, several growth parameters differ between ecotypes of SDTF and BAF (Fig. 4). For example, the male shoots from the SDTF presented a faster development than the female SDTF. In contrast, the male shoots from the BAF had the opposite observed.

In SDTF, our results suggest an advantage for female shoots. For protonema growth in the BAF ecotype, females had a lower T‐med (3.85) than males (4.56), indicating greater protonemal area at that time and, likely, higher competitiveness. For shoot production in BAF, however, the pattern reversed, with females showing a higher T‐med. Regardless, the higher shoot carrying capacity (K) in females suggests greater competitiveness. When male and female plants grow together, females may initially occupy more protonemal area, restricting male development and promoting female‐biased populations, a pattern also reported for *Ceratodon purpureus (Hedw.) Brid*. (Eppley *et al*. 2018). Sex‐based competitiveness in bryophytes remains little studied, but existing work consistently shows female bias: *Sphaerocarpos texanus* Aust. (McLetchie 1992), *C. purpureus* (Shaw *et al*. 1999), *Marchantia inflexa* Nees & Mont. (McLetchie & Puterbaugh 2000) and *W. jamaicensis* (dos Santos *et al*. 2022b) all exhibit greater female performance. In *Hylocomium splendens (Hedw.) Schimp*., sporophytic shoots grow less than sterile ones, reflecting reproductive costs (Rydgren & Økland 2002). Together, these patterns show that sex and environment shape life‐history variation in *B. argenteum*. These patterns indicate that local conditions influence reproductive strategies in *B. argenteum*. We found consistent ecotype‐ and sex‐specific differences in life‐history traits, with BAF and SDTF shoots showing distinct profiles. The faster growth and stronger sexual investment of SDTF shoots may also contribute to the female‐biased patterns reported in natural populations (Glime & Bisang 2017).

### Sex expression is more evident in cultures of SDTF ecotype and differs from what is observed in the field

Absolute and relative sex expression were highest in SDTF males (Fig. 5), a surprising result given that male sex expression is rarely observed in the field (Pôrto *et al*. 2017; Castetter *et al*. 2019). We expected low expression in males, as seen in the BAF ecotype. One explanation involves sex‐specific reproductive costs. In *S. caninervis*, Stark *et al*. (2000) showed that males allocate more to reproduction, supporting the idea that the more costly sex expresses less, a pattern also found in *W. jamaicensis* by Santos *et al*. (2022). However, Bisang *et al*. (2006) reported the opposite in *Pseudocalliergon trifarium*, indicating that this relationship is not universal. Our results similarly suggest that reproductive cost and sex‐expression patterns vary with environment, even within a single species.

Another key point is the trade‐off between sex expression and bulbil production. Sexual and asexual reproduction contribute differently to colonization: spores support long‐distance dispersal, while vegetative propagules favour local establishment (Glime & Bisang 2017; Vigalondo *et al*. 2019). Vegetative propagules can also outperform spores in both occupied and uncolonized areas (Newton & Mishler 1994). Because these modes differ in dispersal and establishment, populations in distinct ecosystems may show contrasting demographic and genetic patterns. This link between reproduction and genetic structure has been demonstrated in other bryophytes. Wang *et al*. (2012), for example, found higher genetic diversity in island populations of the mainly asexual *Hypnum plumaeforme Wilson*., whereas the predominantly sexual *Pogonatum inflexum* (Lindb.) Sande Lac. showed similar diversity across habitats. These cases illustrate how reproductive mode shapes genetic variation, suggesting that SDTF and BAF ecotypes of *B. argenteum* may also differ genetically given their contrasting reliance on sexual reproduction.

### The difference in the development of gametangia may indicate adaptation in the studied ecotypes

The timing of male and female phenophases differed between ecotypes. In SDTF, males initiated gametangia development earlier than females, whereas in BAF the pattern was reversed. At maturity, both ecotypes showed protogyny, but BAF shoots reached this stage about a week later than SDTF. These differences likely reflect ecological adaptation: SDTF plants must complete reproduction within a 4–5‐month window, while BAF plants have year‐round moisture to support longer reproductive periods. More broadly, our findings align with patterns in bryophytes from tropical regions, where gametangia typically develop faster than in temperate species (Glime 2017). In the United Kingdom, for example, *B. argenteum* develops its male gametangia (from sex expression to maturation) over 6–8 months compared to 2–3 months for females (Miles *et al*. 1989). In Brazil, although no phenological studies have been carried out with *B. argenteum*, climate patterns suggest that reproduction is faster compared to the findings of Miles *et al*. (1989) since the limiting factor for the reproduction of tropical species is the rain which in several Brazilian ecosystems are strongly seasonal (Maciel‐Silva & De Oliveira 2016). Of the species studied phenologically from the tropics, all developed over shorter periods (Miles *et al*. 1989; Fotoba 1998; Oliveira & Porto 2001; Pôrto & de Oliveira 2002; Maciel‐Silva *et al*. 2012; dos Santos *et al*. 2020). On the other hand, species from temperate and polar regions present the development of gametangia for a long time. For example, in some species of *Ptychomitrium* in Japan, Deguchi and Takeda (1986) showed that antheridia typically required 9 months, whereas archegonia only needed 1 month to develop. We can interpret that species from the tropical region are adapted to develop more quickly, and consequently, this pattern can vary between the ecotypes found in different tropical ecosystems, which can be affected by geographical distance.

## CONCLUSION

This study shows that *B. argenteum* ecotypes differ in life‐history traits across ecosystems. SDTF plants developed faster and expressed sex more often, while BAF plants showed slower dynamics, consistent with adaptation to contrasting seasonal pressures. We also detected a trade‐off between sexual and asexual reproduction, with possible consequences for genetic structure. Although based on a single case, the results highlight the importance of ecotype–environment interactions. Broader sampling and tests of stress tolerance, genetic structure and vegetative traits are needed to clarify how environment shapes this species.

## AUTHOR CONTRIBUTIONS

WLdS, WL, and FP conceived the study and developed the research questions. WLdS, JG and AM‐R conducted the experiments and collected the data. WLdS performed the statistical analyses and wrote the first draft of the manuscript. WLdS, FP, KCP and LRS contributed to data interpretation and critically revised the manuscript. All authors read and approved the final version of the manuscript.

## Supporting information


**File S1.** Results of generalized linear models (GLMs) testing the effects of sex and ecosystem on protonemal growth, shoot production and sexual expression.

**File S2.** Binomial negative residuals graphics of relationship between bulbil production and sexual expression in *Bryum argenteum*. Each section corresponds to a specific response variable modelled in the study.

## Data Availability

The data that support the findings of this study are openly available in Files Manuscript: Ecological divergence in the silver moss B at https://figshare.com/account/articles/28610258, reference number https://doi.org/10.6084/m9.figshare.28610258.
