# Supplementary material for: Ecological divergence in the silver moss Bryum argenteum: developmental, ontogenetic and life‐history trait variations across contrasting tropical ecosystems
Source: Plant Biol (Stuttg). 2026 Mar 11;28(4):1289–99. doi: 10.1111/plb.70200 (PMC13175951; doi:10.1111/plb.70200)
Supplement: Supplementary file 1 — File S1. Results of generalized linear models (GLMs) testing the effects of sex and ecosystem on protonemal growth, shoot production and sexual expression. [file PLB-28-1289-s001.docx]

**GLM – PROTONEMA**

**
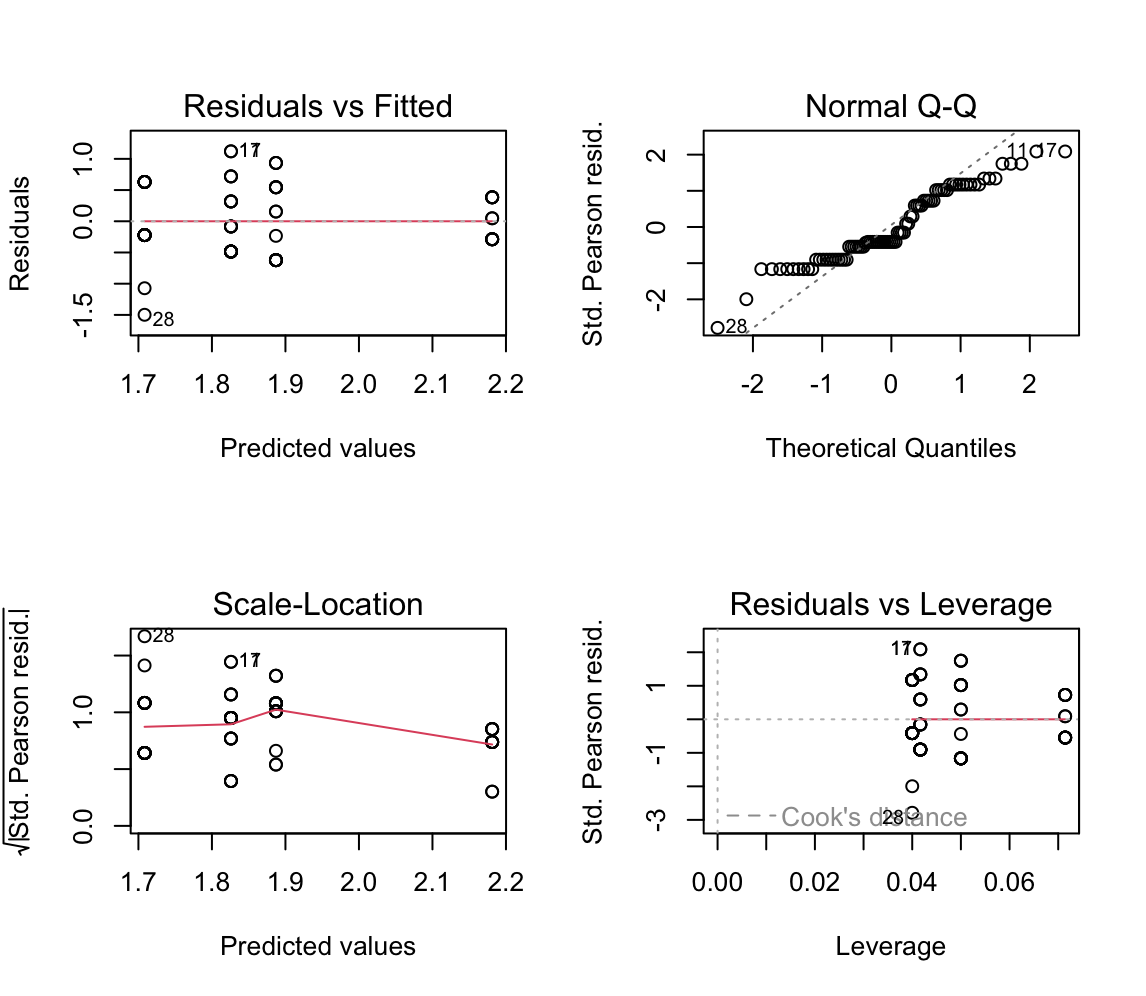
**

**Diagnostic plots of the quasi-Poisson GLM fitted for protonema development in Bryum argenteum.**
Panels show (a) residuals vs. fitted values, (b) normal Q–Q plot, (c) scale–location plot, and (d) residuals vs. leverage, indicating no major violations of model assumptions.

**GLM – SHOOT**

**
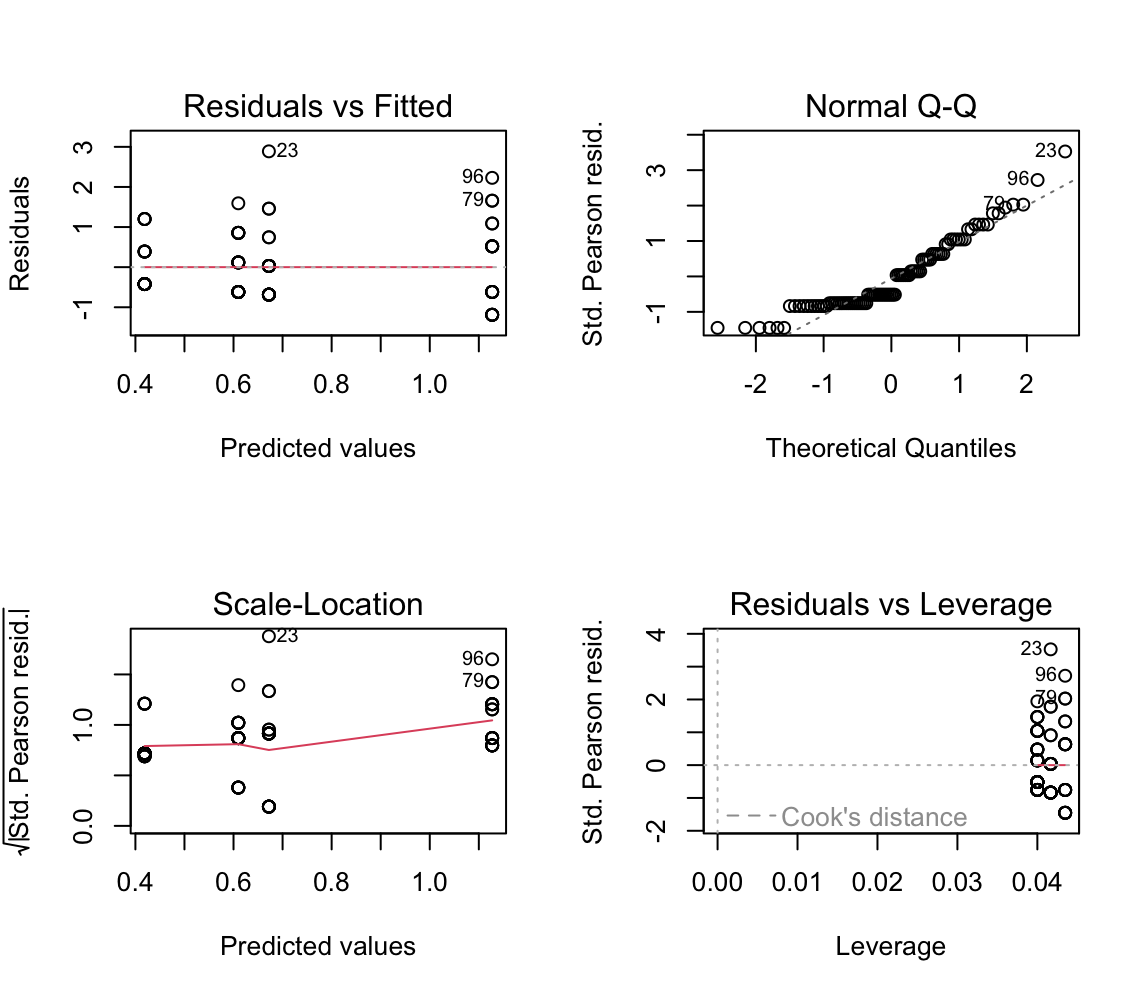
**

**Diagnostic plots of the quasi-Poisson GLM fitted for shoot production in Bryum argenteum.**
Panels show (a) residuals vs. fitted values, (b) normal Q–Q plot, (c) scale–location plot, and (d) residuals vs. leverage, indicating adequate model fit.

**GLM – SEX EXPRESSION**

**
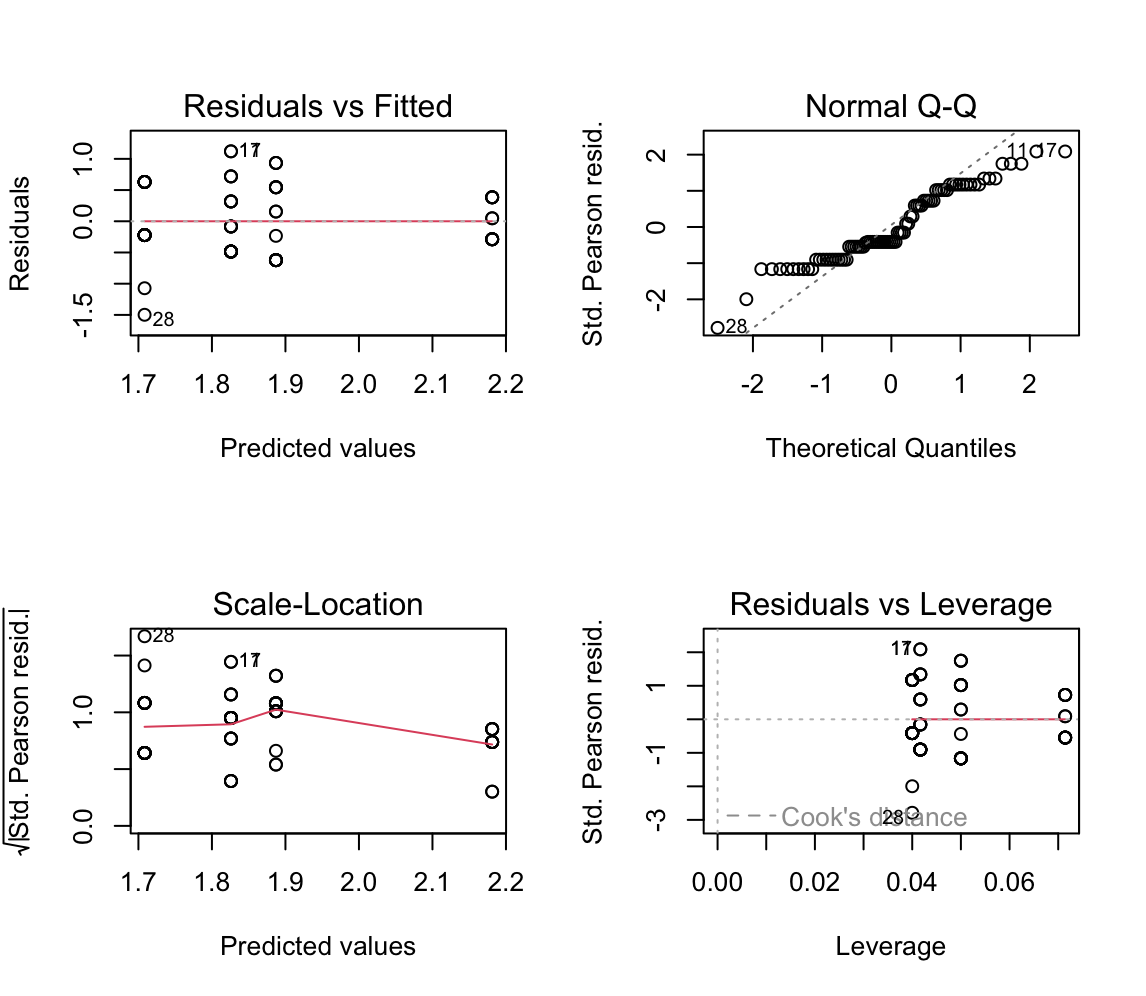
**

**Diagnostic plots of the quasi-Poisson GLM fitted for sex expression in Bryum argenteum.**
Panels show (a) residuals vs. fitted values, (b) normal Q–Q plot, (c) scale–location plot, and (d) residuals vs. leverage, indicating no major deviations from model assumptions.
