# Supplementary material for: Ecological divergence in the silver moss Bryum argenteum: developmental, ontogenetic and life‐history trait variations across contrasting tropical ecosystems
Source: Plant Biol (Stuttg). 2026 Mar 11;28(4):1289–99. doi: 10.1111/plb.70200 (PMC13175951; doi:10.1111/plb.70200)
Supplement: Supplementary file 2 — File S2. Binomial negative residuals graphics of relationship between bulbil production and sexual expression in Bryum argenteum. Each section corresponds to a specific response variable modelled in the study. [file PLB-28-1289-s002.docx]

**###############ANALYSIS 1: Absolute biomass vs. bulbil production ###############**


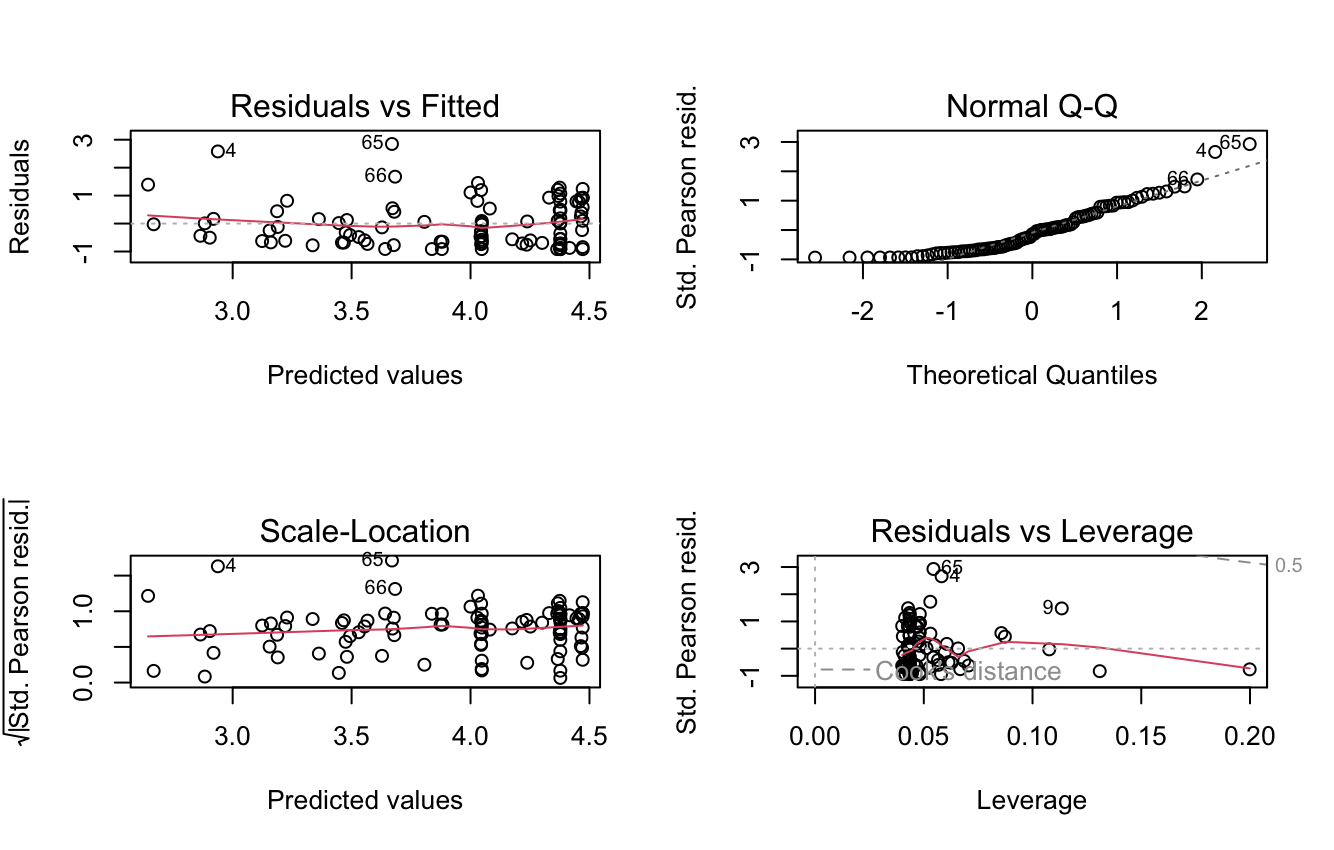


**Diagnostic plots of the negative binomial model relating absolute biomass to bulbil production in Bryum argenteum.**
Panels show (a) residuals vs. fitted values, (b) normal Q–Q plot, (c) scale–location plot, and (d) residuals vs. leverage, indicating an adequate model fit and no strong violations of model assumptions.

**############ANALYSIS 2:Relative repr. Allocatrion vs. bulbil production ############**


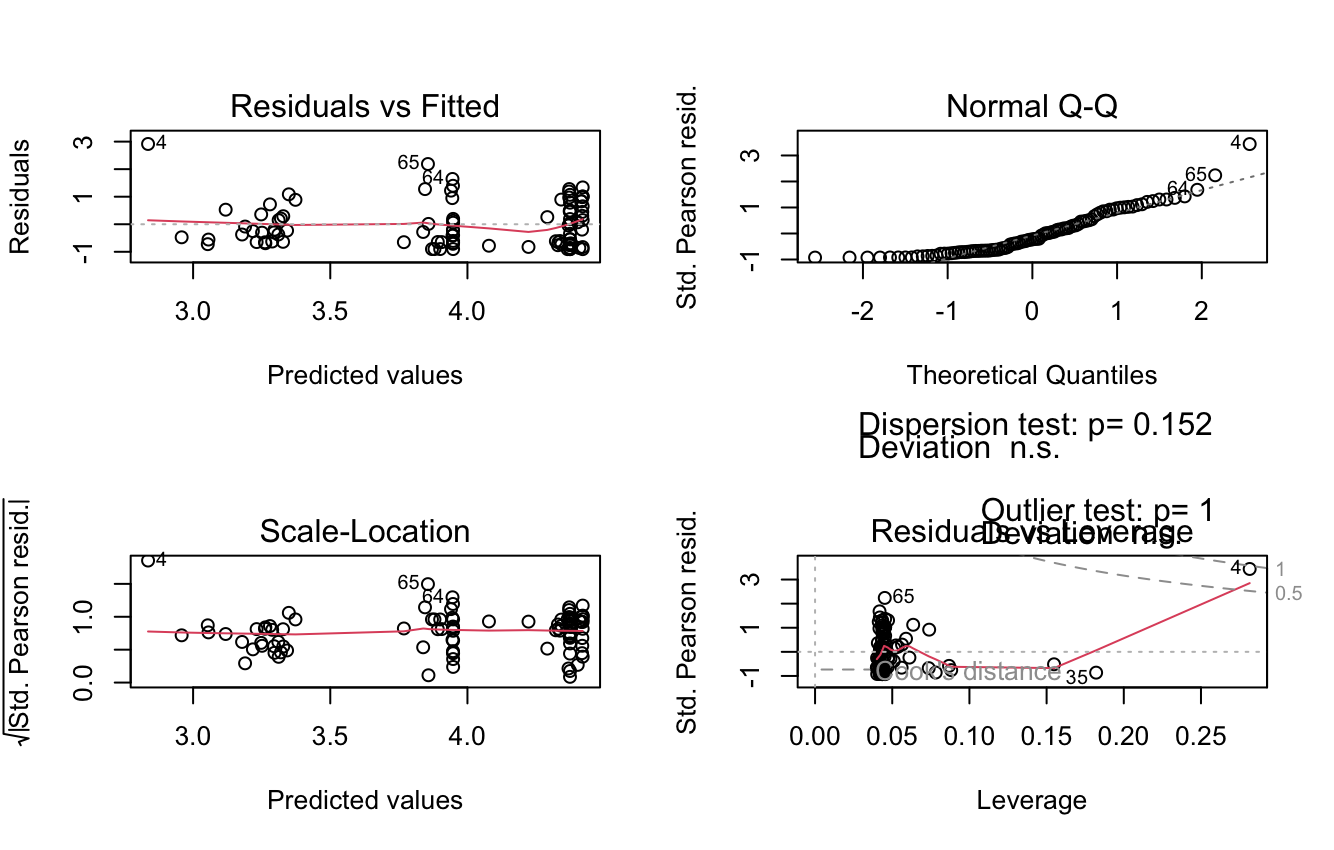


**Diagnostic plots of the negative binomial model relating relative reproductive allocation to bulbil production in Bryum argenteum.**
Panels show (a) residuals vs. fitted values, (b) normal Q–Q plot, (c) scale–location plot, and (d) residuals vs. leverage, indicating no evidence of overdispersion or influential outliers.
